# The Potential of Microbiota-Modulating Dietary Strategies in Gallstone Prevention: Evidence from 2 Large Population-Based Cohorts

**DOI:** 10.5152/tjg.2026.26355

**Published:** 2026-07-22

**Authors:** Hongyan He, Luohong Li, Xiaoli Yang

**Affiliations:** Department of Gastroenterology and Hepatology, West China Hospital, Sichuan University, Chengdu, China

**Keywords:** Cholelithiasis, cross-sectional studies, diet, gastrointestinal microbiome, gallstones, risk factors

## Abstract

**Background/Aims:**

Gut–microbiota dysbiosis has been implicated in gallstone formation through alterations in bile acid and cholesterol metabolism, but whether adherence to a microbiota-supportive diet is associated with gallstone disease at the population level remains unclear. The current study examined the cross-sectional association between the Dietary Index for Gut Microbiota (DI-GM) and the prevalence of gallstone disease.

**Materials and Methods:**

The current study analyzed National Health and Nutrition Examination Survey (NHANES) 2017-2020 (n = 7198 adults aged ≥20 years) and the UK Biobank (n = 126 478) cross-sectionally. DI-GM was calculated using 14 food components in NHANES and 13 food components in the UK Biobank. Gallstone disease was ascertained by self-report in NHANES and by self-report supplemented with inpatient ICD-10 K80 codes in the UK Biobank. The authors used logistic regression (survey-weighted for NHANES and unweighted for the UK Biobank) and restricted cubic splines. A demographic and behavioral adjusted model estimated the total effect and a cardiometabolic adjusted model was used as a conservative sensitivity analysis.

**Results:**

Higher DI-GM scores were associated with lower odds of prevalent gallstones in both cohorts. In the total-effect model, each 1-point increment in the DI-GM score was associated with 13% lower odds of gallstone disease in NHANES (odds ratio (OR) = 0.87, 95% confidence interval (CI): 0.83-0.92) and 7% lower odds in the UK Biobank (OR = 0.93, 0.91-0.95). These associations were attenuated but remained significant after cardiometabolic adjustment (NHANES 0.92, 0.87–0.98; UK Biobank 0.95, 0.93-0.97; both *P* ≤ .007). Compared with the lowest DI-GM category, the highest category was associated with lower odds of gallstone disease in both NHANES (OR, 0.73; 95% CI, 0.60-0.89) and the UK Biobank (OR, 0.80; 95% CI, 0.74-0.87). Restricted cubic spline analyses supported a linear association, and the subgroup and sensitivity analyses yielded consistent results.

**Conclusion:**

In 2 demographically distinct populations, greater adherence to a gut-microbiota-supportive diet was independently associated with lower odds of prevalent gallstone disease. These cross-sectional findings require prospective confirmation before causal or preventive inferences are drawn.

**Main Points**

- The Dietary Index for Gut Microbiota (DI-GM) is a mechanistically based, externally validated index that quantifies the extent to which an individual’s habitual diet supports gut microbial diversity, based on food components with established effects on the gut microbiota.
- Across 2 distinct national datasets (NHANES, N = 7198; UK Biobank, N = 126 478), each 1-point increase in the DI-GM was associated with lower odds of prevalent gallstone disease, and the association remained significant after adjustment for demographic, behavioral, and dietary factors.
- This inverse association demonstrated a linear dose–response shape and remained directionally consistent across sensitivity analyses and participant subgroup analyses in both populations.
- Because of the cross-sectional design, these findings should be interpreted as associative rather than causal. Nevertheless, microbiota-aware dietary indices such as DI-GM may be useful tools for investigating the role of diet–microbiota interactions in biliary health.

## Introduction

Cholelithiasis is a common digestive disorder, with a prevalence of approximately 15% in high-income countries, and remains a leading cause of hospitalization and abdominal surgery worldwide.^1^ In addition to well-established non-modifiable risk factors, such as female sex, older age, and genetic predisposition, and modifiable cardiometabolic risk factors, such as adiposity and metabolic dysregulation, accumulating evidence suggests that behavioral factors, particularly dietary patterns, contribute to disease onset.^2^^,^^3^ However, traditional dietary quality indices were not designed to reflect the mechanistic pathways by which dietary intake influences gallstone formation, particularly the contribution of the gut microbiota.^4^^,^^5^

Accumulating evidence identifies the gut microbiota as a key regulator of bile acid metabolism, lipid metabolism, and enterohepatic circulation all of which are closely involved in the pathogenesis of cholelithogenesis.^6^^,^^7^ Certain bacterial taxa mediate bile acid deconjugation and biotransformation, thereby influencing cholesterol nucleation and susceptibility to gallstone formation.^8^ Conversely, dysbiotic shifts have been observed with altered bile acid profiles that promote gallstone formation.^9^ Because dietary intake strongly influences both the composition and metabolic output of the gut community, dietary patterns that support a diverse microbial community may help maintain bile composition equilibrium and reduce lithogenic risk.^10^

Against this backdrop, the Dietary Index for Gut Microbiota (DI-GM) has been proposed and externally validated as an index that captures the microbiota-supportive quality of a person’s habitual intake.^11^ The DI-GM differs from generic dietary quality indices by incorporating food components with documented effects on microbial composition and downstream metabolic activity, thereby providing a microbiota-oriented framework for dietary assessment. It should be noted, however, that DI-GM is a dietary proxy and does not directly assess the gut microbiome.

To date, although gallstone-related microbial signatures have been identified in clinical studies, population-based studies examining the association between gut microbiota–supportive dietary patterns and gallstone disease burden are limited.^12^ Clarifying this question could improve the understanding of how the diet–microbe axis intersects with biliary pathology. Therefore, the current study examined the cross-sectional association between DI-GM and prevalent gallstone disease in 2 large and demographically distinct populations by analyzing data from the National Health and Nutrition Examination Survey (NHANES) in the United States and the UK Biobank in the United Kingdom, thereby enabling cross-population replication and enhanced statistical resolution.

## Materials and Methods

### Study Population

**Figure 1.**
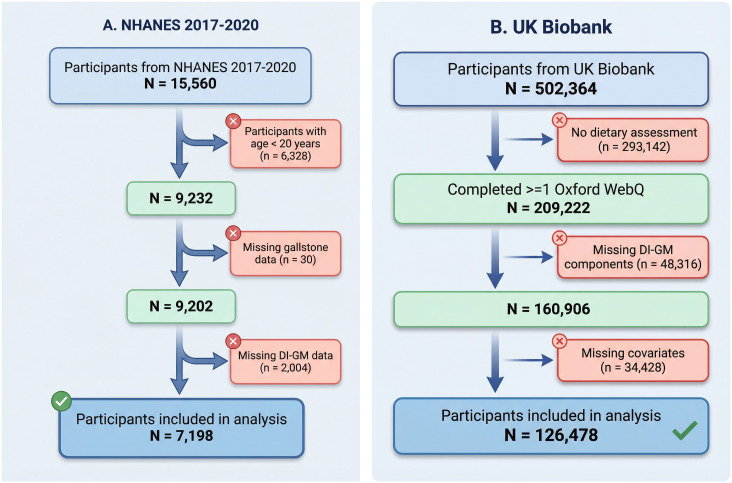
Participant selection flow diagram for (A) the National health and nutrition examination survey (NHANES) 2017-2020 and (B) the United Kingdom (UK) Biobank.

Cross-sectional data were assembled from 2 independent population sources. The first dataset comprised the NHANES 2017-2020 cycle, a nationally representative survey of the civilian, noninstitutionalized United States population that uses a multistage stratified sampling design.^13^ The second dataset was the UK Biobank, a prospective resource comprising more than half a million UK residents aged 37 to 73 years at recruitment between 2006 and 2010.^14^^,15^ Eligibility for the present study required age 20 years or older (NHANES) and complete records for dietary measurement, gallstone outcome, and key covariates. After exclusions for incomplete data, the analytic samples comprised 7198 NHANES participants and 126 478 UK Biobank participants, as detailed in Figure 1.

Both source studies obtained the required ethical approvals (NCHS Research Ethics Review Board for NHANES; North West Multi-center Research Ethics Committee, reference 11/NW/0382, for the UK Biobank). Each participant provided written consent at enrollment into the parent study. Because this secondary analysis used deidentified data, additional ethics committee approval nor informed consent was not required. The authors report findings in accordance with the Strengthening the Reporting of Observational Studies in Epidemiology (STROBE) guideline for cross-sectional studies.^16^

### Gallstone Assessment

Gallstone status in NHANES was determined through the medical conditions module, which asks respondents whether a health care professional has ever told them they had gallstones. Because this item captures a lifetime (“ever told”) history of gallstones, it identifies prevalent disease, including most participants who subsequently underwent cholecystectomy for symptomatic gallstones; imaging confirmation was not available. In the UK Biobank, gallstone status was ascertained using 2 sources. At the baseline assessment, participants reported physician-diagnosed conditions during a nurse-led verbal interview (self-reported noncancer illness data field 20002; code 1162 indicates gallstones/cholelithiasis). In addition, linked hospital inpatient records were searched for the ICD-10 code K80 (cholelithiasis). Participants meeting either criterion were classified as prevalent cases.^17^ Because hospital linkage includes records both before and after the dietary assessment, the primary UK Biobank definition includes prevalent cases and some incident cases. A sensitivity analysis restricted to incident K80 diagnoses recorded after dietary assessment is presented in Supplementary Table 1.

### Definition of the Dietary Index for Gut Microbiota

DI-GM scoring followed the validated framework that quantifies the extent to which habitual dietary intake promotes gut microbial health.^11^^,^^18^ The index comprises food items categorized as a microbiota-favorable arm (including fiber-rich plants, whole grains, broccoli, fermented dairy, and avocado, among others) and a microbiota-unfavorable arm (including red and processed meats, refined cereals, and a high-fat diet). For all components except the high-fat component, sex-specific cohort medians were used as cutoffs for dichotomization, with intakes consistent with microbial benefit assigned 1 point. Consistent with the original index, the high-fat component used a fixed threshold of ≥40% of energy from fat.

In NHANES, individual foods, including green tea, avocado, and cranberries, were identified at the food-code level using the Food and Nutrient Database for Dietary Studies (FNDDS) underlying the 2 24-hour dietary recalls, which permits green tea to be distinguished from other teas. Accordingly, the primary NHANES score retained all 14 components (range 0-14). The authors note that the original index omitted green tea in NHANES (range 0-13). To ensure the findings were not dependent on this approach, sensitivity analyses using a 13-component NHANES score (omitting green tea) and a fully harmonized 12-component score (omitting green tea and chickpeas), computed identically in both cohorts, are presented in Supplementary Table 1. Because chickpea intake is not assessed by the Oxford Web-based 24-hour dietary questionnaire (Oxford WebQ), the UK Biobank index comprised 13 components (range 0-13). For several low-prevalence components, the sex-specific median lies at or near zero; therefore, the corresponding indicator effectively contrasted any consumption with no consumption rather than a graded intake. Component-by-component prevalence for both cohorts is presented in Supplementary Table 2. Component definitions and cutoffs are provided in Supplementary Table 3.

### Covariates

Confounders were prespecified on the basis of prior literature documenting their associations with cholelithiasis.^19^^,^^20^ Demographic variables included age, sex, racial/ethnic identification, and educational level. As a measure of socioeconomic status, NHANES analyses used the poverty-to-income ratio, whereas UK Biobank analyses used the Townsend deprivation index. Behavioral variables included smoking status and alcohol consumption. Clinical variables included measured body mass index (kg/m^2^), self-reported hypertension, diabetes, prior cancer, and circulating high-density lipoprotein cholesterol (HDL-C), and total cholesterol (TC). Nutritional variables included total daily intake of energy, protein, and fat. Because BMI, HDL-C, TC, diabetes, and hypertension plausibly lie on the diet→microbiota→bile-acid/lipid→gallstone pathway, these variables were treated as potential mediators rather than confounders in the primary (total-effect) model (see the section “Statistical Analysis”). Additional sensitivity analyses considered physical activity in both cohorts and, among women, parity and exogenous hormone use in the UK Biobank.

### Statistical Analysis

Continuous variables are reported as means with SDs (or as medians with interquartile ranges for skewed variables), and categorical variables are reported as counts with column percentages. Distributional assumptions were assessed graphically using histograms and Q–Q plots. Group comparisons were performed using the Welch’s *t-*test for approximately normally distributed variables the Mann–Whitney *U-*test for skewed variables, and the Pearson’s *χ*^2^ test for proportions. The NHANES sampling design (stratification, clustering, and unequal selection probabilities) was incorporated using the 2-day dietary sample weight (WTDR2DPP), Taylor-series linearization, and the masked design variables SDMVSTRA (strata) and SDMVPSU (primary sampling units) in accordance with the following NCHS analytic guidelines for the combined January 2017 to March 2020 pre-pandemic data file. The UK Biobank, which is not a probability sample, was analyzed without weighting. The 2 cohorts were not pooled but were analyzed as independent replication cohorts.

The authors’ target estimand was the total effect of habitual DI-GM on gallstone prevalence. Cohort-specific logistic regression models were used to estimate odds ratios and 95% CIs, with DI-GM modeled as both a continuous variable and cohort-specific ordered categories (defined to preserve adequate cell sizes given the differing DI-GM distributions). Model 1 adjusted for age and sex. Model 2 (the primary, total-effect model) adjusted for race/ethnicity, educational level, socioeconomic status (PIR or Townsend), smoking, and alcohol consumption. Model 3, included as a conservative sensitivity analysis, further adjusted for the putative cardiometabolic mediators (BMI, HDL-C, TC, diabetes, and hypertension), prior cancer, and energy, protein, and fat intake. Because conditioning on intermediates can induce overadjustment bias toward the null, Model 2 was interpreted as the estimate of the total effect, whereas Model 3 was interpreted as the lower-bound estimate. The assumed causal structure and the corresponding minimal adjustment set are shown in Supplementary Figure 1 (directed acyclic graph).^21^^,^^22^ To assess nonlinearity, 4-knot restricted cubic spline curves were fitted with knots at the 5th, 35th, 65th, and 95th percentiles of the DI-GM score and applied identically in both cohorts. Effect modification was examined by stratifying analyses according to age, sex, race/ethnicity, educational level, socioeconomic status, BMI, and hypertension. Tests for subgroup interactions were exploratory and were not adjusted for multiple comparisons. Robustness was evaluated through prespecified sensitivity analyses (Supplementary Table 1). Consistent with the cross-sectional design, results are described as associations rather than effects.^23^ All *P* values were 2-sided, and *P* < .05 was considered statistically significant. All analyses were performed using *R* version 4.3.0.

## Results

### Baseline Characteristics

Participant profiles for the 2 cohorts are summarized in Table 1. In NHANES, 7198 adults (mean age 50.8 years) were included, of whom 782 (10.9%) reported a previous diagnosis of gallstones. The UK Biobank sample included 126 478 adults (mean age 56.8 years), of whom 11 640 (9.2%) had gallstone disease. In both cohorts, participants with gallstones were older, were more likely to be women, and had greater adiposity and a higher prevalence of diabetes and hypertension than those without gallstones (*P* < .001 for each). For DI-GM, the crude unweighted NHANES means were nearly identical between participants without gallstones and with gallstones (4.50 vs. 4.48). After survey weighting, the gallstone group had a modestly lower mean DI-GM score (4.3 vs. 4.5). In the UK Biobank, participants with gallstones had a lower mean DI-GM score than those without gallstones (4.82 vs. 5.15; *P* < .001). The near-identical crude NHANES mean DI-GM scores reflect strong negative confounding. Participants with gallstones were substantially older and more likely to be women characteristics positively associated with both gallstone prevalence and, in this cohort, slightly higher DI-GM scores. Accordingly, the inverse association became apparent only after adjustment for age, sex, and socioeconomic status.

### DI-GM Score and Odds of Gallstone Disease

**Figure 2.**
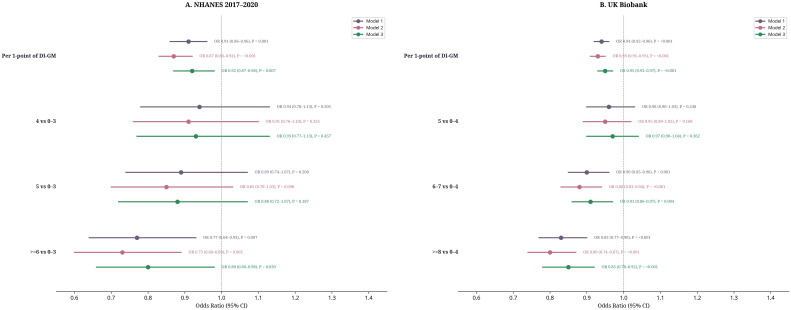
Odds ratios (95% confidence interval) for prevalent gallstone disease per 1-point increase in the dietary index for gut microbiota (DI-GM) and across DI-GM categories, by cohort and adjustment model (models 1-3). (A) National health and nutrition examination survey (NHANES); (B) United Kingdom (UK) Biobank.

In both cohorts, higher DI-GM scores were associated with lower odds of gallstones (Table 2 and Figure 2). In the primary total-effect model (Model 2), each 1-point increase in the DI-GM score was associated with 13% lower odds of gallstones in NHANES (OR = 0.87, 95% CI: 0.83-0.92; *P* < .001) and 7% lower odds in the UK Biobank (OR = 0.93, 95% CI: 0.91-0.95; *P* < .001). Estimates were attenuated but persisted in the conservative mediator-adjusted model (Model 3): NHANES: OR = 0.92; 95% CI: 0.87-0.98; *P* = .007; UK Biobank: OR = 0.95; 95% CI: 0.93-0.97; *P* < .001. This pattern was consistent with partial mediation through cardiometabolic factors.

**Figure 3.**
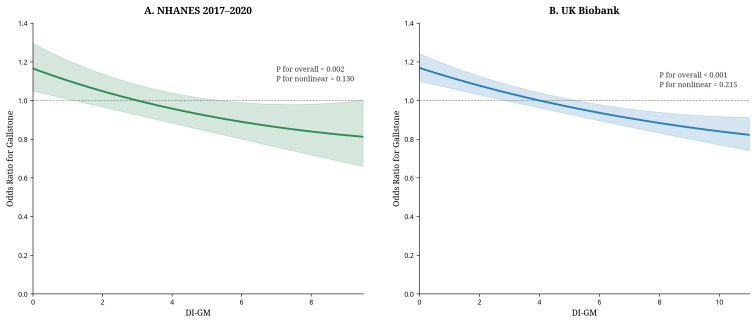
Restricted cubic spline dose–response curves (4 knots at the 5th, 35th, 65th, and 95th percentiles) for the association between dietary index for gut microbiota (DI-GM) score and the odds of prevalent gallstone disease. (A) National health and nutrition examination survey (NHANES); (B) United Kingdom (UK) Biobank. Shaded areas denote 95% CIs.

Categorical analyses reinforced this pattern. Compared with the lowest DI-GM category, the highest category was associated with lower odds of gallstones in the primary total-effect model (Model 2) in both NHANES (OR = 0.73, 95% CI: 0.60-0.89) and the UK Biobank (OR = 0.80, 95% CI: 0.74-0.87). The conservative model demonstrated a similar pattern in both NHANES (OR = 0.80, 0.66-0.98) and the UK Biobank (OR = 0.85, 0.78-0.92). Spline-based dose–response evaluation demonstrated a linear, downward trajectory in both cohorts (*P* for the overall association = .002 in NHANES and < .001 in the UK Biobank; *P* for nonlinearity = .13 and .22, respectively) (Figure 3). On the absolute scale, the model-standardized predicted prevalence of gallstones was 2.1 percentage points lower in the highest than in the lowest DI-GM category in NHANES (95% CI: −3.4 to −0.8) and 1.4 percentage points lower in the UK Biobank (95% CI: −1.9 to −0.9), indicating that the per-point association, although consistent, was modest.

### Subgroup and Sensitivity Analyses

**Figure 4.**
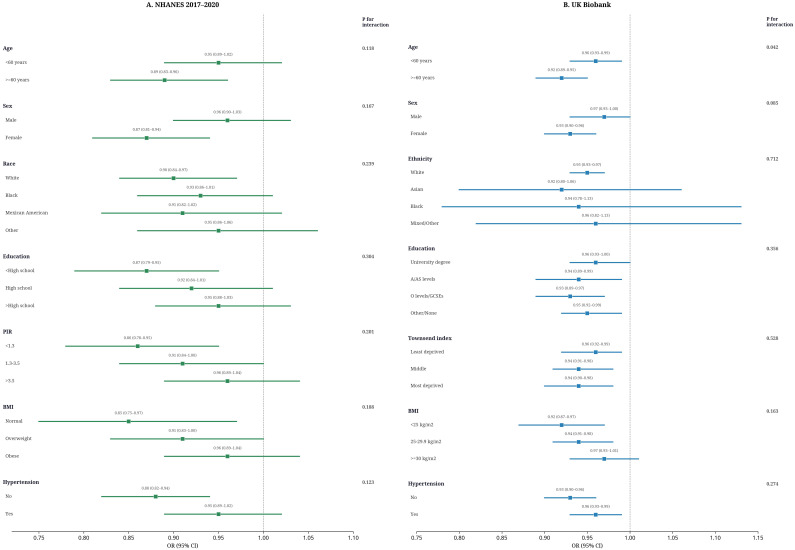
Subgroup analyses of the association between dietary index for gut microbiota (DI-GM) (per 1-point) and prevalent gallstone disease, with *P* for interaction. (A) National health and nutrition examination survey (NHANES); (B) United Kingdom (UK) Biobank.

Stratified analyses indicated that the inverse DI-GM association extended broadly across demographic and clinical subgroups within each dataset (Figure 4). Two nominal interactions were observed: age in the UK Biobank (*P*-interaction = .04), with somewhat stronger inverse associations among older participants, and educational level in NHANES (*P*-interaction = .008). Given the number of exploratory tests and the absence of adjustment for multiple comparisons, these findings should be interpreted cautiously and considered hypothesis generating. No significant interactions were observed for sex, BMI, or comorbid metabolic disease. The association appeared somewhat weaker among participants with higher metabolic risk (e.g., obesity, hypertension).

Sensitivity testing (Supplementary Table 1) was consistent with these estimates. Effect sizes were the same even after exclusion of adults with diabetes, those with cancer, or those with extreme energy intake and after multiple imputation of missing covariates. In the UK Biobank, restricting the outcome to hospital-confirmed K80 episodes also reproduced the inverse association (OR = 0.94, 95% CI: 0.91-0.97). Additional sensitivity analyses using the 13-component and harmonized 12-component scores, per-SD standardized DI-GM scores, exclusion of participants who had undergone cholecystectomy, additional adjustment for physical activity and reproductive factors, restriction to incident UK Biobank cases, and restriction to participants with at least 2 Oxford WebQ recalls are presented in Supplementary Table 1 and were directionally consistent with the main analysis (e.g., the harmonized 12-component score yielded an OR of 0.93 (95% CI: 0.87-0.99) in NHANES and 0.96 (95% CI: 0.94-0.98) in the UK Biobank.

## Discussion

Using data from 2 large, independent populations, the authors observed that a higher DI-GM score, reflecting greater adherence to a microbiota-favorable diet, was associated with lower odds of prevalent gallstone disease in a consistent, inverse, and dose-dependent manner. The association remained after progressive adjustment for demographic background and behavioral exposures, was attenuated but persisted after additional adjustment for cardiometabolic factors, and demonstrated a near-linear gradient across the score range. Because both analyses were cross-sectional, these results should be interpreted as associations rather than evidence of a preventive effect.

Diet–gallstone relationships have been examined previously, but most studies have relied on broad dietary quality scores or individual nutrient classes.^16^^,^^24^^,^^25^ Collectively, this prior literature attributes a favorable signal to plant-rich, high-fiber, unsaturated-fat patterns, alongside an adverse signal from saturated-fat, cholesterol, and refined carbohydrate-heavy diets.^26^^,^^27^ What these traditional metrics typically leave unaddressed is the link between dietary intake and microbial ecology. By using a microbiota-informed dietary index, the authors explicitly captured food components with documented effects on microbial composition and metabolic output.^28^ The authors do not claim that DI-GM score outperforms established dietary quality indices, with which it is moderately correlated (in NHANES, Spearman *r* = 0.42 with the HEI-2020). After mutual adjustment for HEI-2020, the DI-GM scores remained inversely associated with gallstones (OR 0.94, 95% CI 0.88-1.00). Rather, the DI-GM score provides a mechanism-oriented, microbiota-focused perspective that demonstrated a consistent inverse association across 2 cohorts.

Several biological pathways may underlie this association. First, gut commensals catalyze the deconjugation and structural modification of bile acids, with downstream effects on bile composition and cholesterol solubility.^29^^,^^30^ Disrupted microbial communities may shift the bile acid pool toward more hydrophobic species that favor cholesterol nucleation and stone formation.^31^ Experimental studies have shown that transplantation of gut microbiota from patients with gallstones can induce stone formation in mice, with enrichment of Desulfovibrionales increasing bile-acid hydrophobicity and biliary cholesterol secretion,^7^ providing direct support for a diet–microbiota–bile-acid–stone pathway. Second, reduced gut bacterial richness has been repeatedly reported among people with gallstone disease, and low-diversity ecosystems may struggle to maintain bile acid balance and barrier integrity.^9^^,^^32^^,^^33^^,^^34^^,^^35^ Fiber-rich, plant-based dietary patterns—precisely the patterns rewarded by DI-GM—promote microbial richness and taxa relevant to bile metabolism.^36^ Third, microbial metabolites influence hepatic cholesterol metabolism.^37^^,^^38^ Importantly, the authors did not measure the gut microbiome (e.g., by 16S rRNA or metagenomic sequencing). The DI-GM score should be regarded as a dietary proxy, and these mechanisms are therefore plausible, externally supported hypotheses rather than findings of the present study.

A further consideration is the direction of inference. Because diet and prevalent gallstone status were ascertained contemporaneously, reverse causation is plausible. A diagnosis of gallstone related symptoms, dietary counseling, or cholecystectomy may itself prompt a shift toward a healthier, higher-DI-GM diet, which would generate an inverse association in the absence of any protective effect. Two features temper but do not eliminate this concern. In the UK Biobank, the inverse association persisted when the outcome was restricted to hospital-coded disease, and a sensitivity analysis restricted to incident K80 diagnoses recorded after dietary assessment yielded similar results (OR = 0.96, 95% CI: 0.94-0.98; *P* < .001; 2,297 incident cases among 114 838 participants), arguing against substantial reverse causation in the UK Biobank. For NHANES, which is intrinsically cross-sectional, temporality cannot be established, and prospective data are required.

The higher prevalence of diabetes and hypertension among participants with gallstones also warrants consideration. Metabolic syndrome promotes gallstone formation partly through impaired gallbladder motility, autonomic dysfunction, bile stasis, and increased biliary cholesterol saturation. These conditions may act as both confounders and mediators of the association between diet and gallstones, which is why the authors treated them as intermediates in the primary model. The somewhat weaker association observed among participants with higher metabolic risk is consistent with the possibility that, once these pathways are established, dietary quality matters less.

Although both study populations were drawn from Western countries, the findings may be relevant to other populations. Several DI-GM-favorable elements—vegetables, legumes, and pulses (including chickpeas and lentils), whole grains, olive-oil–based unsaturated fat, fermented dairy, and tea and coffee—are characteristic of Mediterranean and many Middle Eastern dietary patterns. Accordingly, the DI-GM score may capture beneficial dietary patterns in these settings. However, because food consumption patterns and component cutoffs vary across populations, direct validation of the DI-GM score in Mediterranean and Middle Eastern cohorts, which are of particular interest to this journal’s readership, is warranted.

From a public-health perspective, cholelithiasis remains widespread and costly.^39^ Dietary modification is comparatively inexpensive and scalable, and if the present associations are confirmed prospectively, microbiota-oriented dietary quality may be one of several modifiable factors associated with gallstone burden. The authors caution against stronger translational claims: the per-point association is modest, the very large UK Biobank sample renders small differences statistically significant, and the association appears attenuated in participants with higher metabolic risk. Accordingly, the authors refrain from recommending DI-GM for individual risk stratification or counseling pending prospective and, ideally, interventional evidence.^40^

Methodological strengths include parallel analyses in 2 large well-characterized datasets, which provided substantial statistical resolution, natural cross-population replication, and use of a mechanism-anchored, validated dietary index alongside thorough confounder control. Several limitations apply. First, the cross-sectional design precludes causal inference, and reverse causation and residual confounding (e.g., by rapid weight loss, which is poorly captured in both cohorts) cannot be excluded. Second, dietary exposure relied on short-window self-reported instruments—24-hour recalls in NHANES and the Oxford WebQ in the UK Biobank—which may not accurately reflect long-term habitual intake relevant to a chronic disease process. Although averaging repeated dietary assessments may reduce exposure misclassification, it does not eliminate it.^41^ Third, because gallstones are frequently asymptomatic, neither cohort underwent systematic ultrasonographic screening, and some participants with undiagnosed gallstones were misclassified as controls. Together with the reliance on self-reported gallstone status and the absence of imaging confirmation in NHANES, this likely resulted in nondifferential outcome misclassification, which would most plausibly bias the association toward the null, rendering the estimates conservative. Fourth, the per-point ORs are not strictly comparable across cohorts because of the differing score range (0-14 vs 0-13); harmonized-score and per-SD analyses are therefore provided. Fifth, the UK Biobank exhibits a well-described healthy-volunteer selection effect, and Oxford WebQ represents a further self-selected subset. Although internal exposure–outcome associations are often broadly generalizable,^15^ absolute prevalence is not, and a comparison of included versus excluded participants is provided (Supplementary Table 4). Sixth, the primary analysis was based on complete-case data. Although missingness was unlikely to be completely at random, the close agreement between multiple-imputation and complete-case analyses argues against substantial bias. Finally, DI-GM comprises a fixed, literature-derived set of components and does not capture the full complexity of habitual dietary intake or microbiota-relevant exposures. In addition, no microbiome data were available to confirm microbial changes.

In summary, across 2 large population-based resources from the United States and the United Kingdom, greater adherence to a microbiota-supportive diet—as measured by the DI-GM—was independently associated with lower odds of prevalent gallstone disease. The association was consistent in direction and magnitude across adjustment models, dose–response analyses, subgroup analyses, and sensitivity analyses. These cross-sectional findings support the role of diet–microbiome interactions in biliary health and provide a rationale for prospective and mechanistic studies before any preventive inference can be made.

## Supplementary Materials

Supplementary Material

## Figures and Tables

**Table 1.** Baseline Characteristics of Participants by Gallstone Status in National Health and Nutrition Examination Survey and the United Kingdom Biobank

Characteristic	NHANES: No Gallstone	NHANES: Gallstone	NHANES, *P*	UKB: No gallstone	UKB: Gallstone	UKB, *P*
N	6416	782	—	114 838	11 640	—
Age, years, mean (SD)	50.4	57.9	<.001	56.3	61.9	<.001
Female, %	49.3	66.5	<.001	52.8	66.0	<.001
BMI, kg/m^2^, mean (SD)	29.4	32.9	<.001	27.0	31.1	<.001
Diabetes, %	13.3	24.0	<.001	4.4	11.0	<.001
Hypertension, %	36.2	54.3	<.001	23.3	41.9	<.001
Current smoking, %	18.1	21.7	<.001	10.2	13.3	<.001
HDL-C / TC	54.2 / 191.4	47.7 / 201.0	<.001	1.5 / 5.7	1.3 / 6.0	<.001
DI-GM, mean (SD)	4.5	4.3	exploratory	5.15	4.82	<.001

Values are mean or %. NHANES estimates are survey-weighted (WTDR2DPP); weighted NHANES totals were mean age 51.2 years, 51.2% female, and 11.1% gallstone prevalence. UK Biobank estimates are unweighted; HDL-C and total cholesterol are reported in mg/dL (NHANES) and mmol/L (UK Biobank).

BMI, body mass index; DI-GM, Dietary Index for Gut Microbiota; HDL-C, high-density lipoprotein cholesterol; TC, total cholesterol; UKB, UK Biobank.

**Table 2.** Association Between Dietary Index for Gut Microbiota and Odds of Prevalent Gallstone Disease in National Health and Nutrition Examination Survey and the United Kingdom Biobank

Exposure	Model 1, OR (95% CI), *P*	Model 2 (Total Effect), OR (95% CI), *P*	Model 3 (Conservative), OR (95% CI), *P*
NHANES 2017-2020			
Per 1-point DI-GM	0.91 (0.86-0.96), .001	0.87 (0.83-0.92), <.001	0.92 (0.87-0.98), .007
Category 4 vs 0-3	0.94 (0.78-1.13), .505	0.91 (0.76-1.10), .325	0.93 (0.77-1.13), .457
Category 5 vs 0-3	0.89 (0.74-1.07), .208	0.85 (0.70-1.03), .098	0.88 (0.72-1.07), .187
Category ≥6 vs 0-3	0.77 (0.64-0.93), .007	0.73 (0.60-0.89), .002	0.80 (0.66-0.98), .030
UK Biobank			
Per 1-point DI-GM	0.94 (0.92-0.96), <.001	0.93 (0.91-0.95), <.001	0.95 (0.93-0.97), <.001
Category 5 vs 0-4	0.96 (0.90-1.03), .248	0.95 (0.89-1.02), .168	0.97 (0.90-1.04), .362
Category 6-7 vs 0-4	0.90 (0.85-0.96), .001	0.88 (0.83-0.94), <.001	0.91 (0.86-0.97), .004
Category ≥8 vs 0-4	0.83 (0.77-0.90), <.001	0.80 (0.74-0.87), <.001	0.85 (0.78-0.92), <.001

Model 1: age, sex. Model 2 (primary, total-effect): + race/ethnicity, education, socioeconomic index (PIR or Townsend), smoking, alcohol. Model 3 (conservative sensitivity): + BMI, HDL-C, TC, diabetes, hypertension, prior cancer, and energy, protein, and fat intake (potential mediators). Reference category is the lowest DI-GM stratum.

BMI, body mass index; CI, confidence interval; DI-GM, Dietary Index for Gut Microbiota; HDL-C, high-density lipoprotein cholesterol; OR, odds ratio; PIR, poverty-to-income ratio; TC, total cholesterol.

## Data Availability

The data that support the findings of this study are available on request from the corresponding author.

## References

[1] Wang X Yu W Jiang G Global epidemiology of gallstones in the 21st century: a systematic review and meta-analysis. Clin Gastroenterol Hepatol. 2024;22(8):1586 1595. (doi: 10.1016/j.cgh.2024.01.051) 38382725

[2] Cuevas A Miquel JF Reyes MS Diet as a risk factor for cholesterol gallstone disease. J Am Coll Nutr. 2004;23(3):187 196. (doi: 10.1080/07315724.2004.10719360) 15190042

[3] Yoo EH Lee SY. The prevalence and risk factors for gallstone disease. Clin Chem Lab Med. 2009;47(7):795 807. (doi: 10.1515/CCLM.2009.194) 19499973

[4] Nie C Yang T Wang Z Dietary patterns and gallstone risks in Chinese adults: a cross-sectional analysis of the China Multi-Ethnic Cohort Study. J Epidemiol. 2023;33(9):471 477. (doi: 10.2188/jea.JE20220039) 35466159 PMC10409532

[5] Wirth J Joshi AD Song M A healthy lifestyle pattern and the risk of symptomatic gallstone disease: results from 2 prospective cohort studies. Am J Clin Nutr. 2020;112(3):586 594. (doi: 10.1093/ajcn/nqaa154) 32614416 PMC7458768

[6] Wahlström A Sayin SI Marschall HU Intestinal crosstalk between bile acids and microbiota and its impact on host metabolism. Cell Metab. 2016;24(1):41 50. (doi: 10.1016/j.cmet.2016.05.005) 27320064

[7] Hu H Shao W Liu Q Gut microbiota promotes cholesterol gallstone formation by modulating bile acid composition and biliary cholesterol secretion. Nat Commun. 2022;13(1):252. (doi: 10.1038/s41467-021-27758-8) PMC8752841 35017486

[8] Park S Kang J Choi S Cholesterol-lowering effect of Lactobacillus rhamnosus BFE5264 and its influence on the gut microbiome and propionate level in a murine model. PLOS One. 2018;13(8):e0203150. (doi: 10.1371/journal.pone.0203150) PMC6112659 30153290

[9] Dan WY Yang YS Peng LH Gastrointestinal microbiome and cholelithiasis: current status and perspectives. World J Gastroenterol. 2023;29(10):1589 1601. (doi: 10.3748/wjg.v29.i10.1589) 36970590 PMC10037248

[10] Le HH Lee MT Besler KR Characterization of interactions of dietary cholesterol with the murine and human gut microbiome. Nat Microbiol. 2022;7(9):1390 1403. (doi: 10.1038/s41564-022-01195-9) 35982311 PMC9417993

[11] Kase BE Liese AD Zhang J The development and evaluation of a literature-based dietary index for gut microbiota. Nutrients. 2024;16(7):1045. (doi: 10.3390/nu16071045) PMC11013161 38613077

[12] Wang Q Jiao L He C Alteration of gut microbiota in association with cholesterol gallstone formation in mice. BMC Gastroenterol. 2017;17(1):74. (doi: 10.1186/s12876-017-0629-2) PMC5466737 28599622

[13] Zhang Y Liu M Xie R. Associations between cadmium exposure and whole-body aging: mediation analysis in the NHANES. BMC Public Health. 2023;23(1):1675. (doi: 10.1186/s12889-023-16643-2) PMC10469832 37653508

[14] Sudlow C Gallacher J Allen N UK Biobank: an open access resource for identifying the causes of a wide range of complex diseases of middle and old age. PLOS Med. 2015;12(3):e1001779. (doi: 10.1371/journal.pmed.1001779) PMC4380465 25826379

[15] Fry A Littlejohns TJ Sudlow C Comparison of sociodemographic and health-related characteristics of UK Biobank participants with those of the general population. Am J Epidemiol. 2017;186(9):1026 1034. (doi: 10.1093/aje/kwx246) 28641372 PMC5860371

[16] Cheng J Zhuang Q Wang W Association of pro-inflammatory diet with increased risk of gallstone disease: a cross-sectional study of NHANES January 2017-March 2020. Front Nutr. 2024;11:1344699. (doi: 10.3389/fnut.2024.1344699) PMC10972905 38549748

[17] Zheng Y Hou J Guo S The association between the dietary index for gut microbiota and metabolic dysfunction-associated fatty liver disease: a cross-sectional study. Diabetol Metab Syndr. 2025;17(1):17. (doi: 10.1186/s13098-025-01589-9) PMC11740478 39825360

[18] Niu Y Xiao L Feng L. Association between dietary index for gut microbiota and metabolic syndrome risk: a cross-sectional analysis of NHANES 2007-2018. Sci Rep. 2025;15(1):15153. (doi: 10.1038/s41598-025-99396-9) PMC12044051 40307409

[19] Wang Y Zhang B Feng L A study of correlation of the dietary index for gut microbiota with non-alcoholic fatty liver disease based on 2007-2018 National Health and Nutrition Examination Survey. Front Nutr. 2025;12:1573249. (doi: 10.3389/fnut.2025.1573249) PMC12018250 40276530

[20] Schisterman EF Cole SR Platt RW. Overadjustment bias and unnecessary adjustment in epidemiologic studies. Epidemiology. 2009;20(4):488 495. (doi: 10.1097/EDE.0b013e3181a819a1) 19525685 PMC2744485

[21] Tennant PWG Murray EJ Arnold KF Use of directed acyclic graphs (DAGs) to identify confounders in applied health research: review and recommendations. Int J Epidemiol. 2021;50(2):620 632. (doi: 10.1093/ije/dyaa213) 33330936 PMC8128477

[22] Hernán MA. The C-word: scientific euphemisms do not improve causal inference from observational data. Am J Public Health. 2018;108(5):616 619. (doi: 10.2105/AJPH.2018.304337) 29565659 PMC5888052

[23] Li L Liu C Xia T Association between plant-based dietary index and gallstone disease: a cross sectional study from NHANES. PLOS One. 2024;19(6):e0305822. (doi: 10.1371/journal.pone.0305822) PMC11198842 38917153

[24] Wu W Pei Y Wang J Association of dietary quality indicators with gallstones in the US: NHANES 2017-2020. BMC Public Health. 2025;25(1):976. (doi: 10.1186/s12889-025-21783-8) 40075394 PMC11905512

[25] Liu T Lv H Li J Association between dietary fiber intake and gallstone disease in US adults: data from NHANES 2017-2020. SLAS Technol. 2025;31:100259. (doi: 10.1016/j.slast.2025.100259) 40024537

[26] Zhang M Yang A. Association between oxidative balance score and gallstone disease: a population-based study from NHANES. Front Nutr. 2025;12:1539969. (doi: 10.3389/fnut.2025.1539969) PMC11796620 39911802

[27] Liu J Liu H Li W Association between dietary index for gut microbiota and self-reported severe headache or migraine in U.S. adults: a cross-sectional study from NHANES. Front Nutr. 2025;12:1549251. (doi: 10.3389/fnut.2025.1549251) PMC11994445 40230722

[28] Grigor’eva IN Romanova TI. Gallstone disease and microbiome. Microorganisms. 2020;8(6):835. (doi: 10.3390/microorganisms8060835) PMC7356158 32498344

[29] Grigor’eva IN. Gallstone disease, obesity and the Firmicutes/Bacteroidetes ratio as a possible biomarker of gut dysbiosis. J Pers Med. 2020;11(1):13. (doi: 10.3390/jpm11010013) PMC7823692 33375615

[30] Ramírez-Pérez O Cruz-Ramón V Chinchilla-López P The role of the gut microbiota in bile acid metabolism. Ann Hepatol. 2017;16(1):S21 S26. (doi: 10.5604/01.3001.0010.5672) 31196631

[31] Cai J Rimal B Jiang C Bile acid metabolism and signaling, the microbiota, and metabolic disease. Pharmacol Ther. 2022;237:108238. (doi: 10.1016/j.pharmthera.2022.108238) 35792223

[32] Staley C Weingarden AR Khoruts A Interaction of gut microbiota with bile acid metabolism and its influence on disease states. Appl Microbiol Biotechnol. 2017;101(1):47 64. (doi: 10.1007/s00253-016-8006-6) 27888332 PMC5203956

[33] Song ST Cai LY Zeng X Gut microbial profile in asymptomatic gallstones. Front Microbiol. 2022;13:882265. (doi: 10.3389/fmicb.2022.882265) PMC9234526 35770155

[34] Tehrani AN Saadati S Yari Z Dietary fiber intake and risk of gallstone: a case-control study. BMC Gastroenterol. 2023;23(1):119. (doi: 10.1186/s12876-023-02752-0) PMC10091554 37041462

[35] Di Ciaula A Portincasa P. Recent advances in understanding and managing cholesterol gallstones. F1000Res. 2018;7:F1000 Faculty Rev-1529. (doi: 10.12688/f1000research.15505.1) PMC6173119 30345010

[36] Portincasa P Moschetta A Palasciano G. Cholesterol gallstone disease. Lancet. 2006;368(9531):230 239. (doi: 10.1016/S0140-6736(06)69044-2) 16844493

[37] Georgescu D Ionita I Lascu A Gallstone disease and bacterial metabolic performance of gut microbiota in middle-aged and older patients. Int J Gen Med. 2022;15:5513 5531. (doi: 10.2147/IJGM.S350104) 35702368 PMC9188808

[38] Nealon WH Urrutia F Fleming D The economic burden of gallstone lithotripsy. Will cost determine its fate? Ann Surg. 1991;213(6):645 9; discussion 649. (doi: 10.1097/00000658-199106000-00015) 2039296 PMC1358595

[39] European Association for the Study of the Liver (EASL). Electronic address: easloffice@easloffice.eu. EASL Clinical Practice Guidelines on the prevention, diagnosis and treatment of gallstones. J Hepatol. 2016;65(1):146 181. (doi: 10.1016/j.jhep.2016.03.005) 27085810

[40] von Elm E Altman DG Egger M The Strengthening the Reporting of Observational Studies in Epidemiology (STROBE) statement: guidelines for reporting observational studies. Lancet. 2007;370(9596):1453 1457. (doi: 10.1016/S0140-6736(07)61602-X) 18064739

[41] Greenwood DC Hardie LJ Frost GS Validation of the Oxford WebQ online 24-hour dietary questionnaire using biomarkers. Am J Epidemiol. 2019;188(10):1858 1867. (doi: 10.1093/aje/kwz165) 31318012 PMC7254925

