# Supplementary material for: The Potential of Microbiota-Modulating Dietary Strategies in Gallstone Prevention: Evidence from 2 Large Population-Based Cohorts
Source: Turk J Gastroenterol. 2026 Jul 22;37(8):880–90. doi: 10.5152/tjg.2026.26355 (PMC13455522; doi:10.5152/tjg.2026.26355)
Supplement: Supplementary file 1 [file supplementary_material.pdf]

Supplementary Table 1. Sensitivity analyses for the association between DI-GM and prevalent gallstone disease

Sensitivity analysis (per 1-point DI-GM)	NHANES OR (95% CI)	P	UK Biobank OR (95% CI)	*P*
Main analysis (conservative model)	0.92 (0.87–0.98)	.007	0.95 (0.93–0.97)	<.001
Primary total-effect model (no metabolic mediators)	0.87 (0.83–0.92)	<.001	0.93 (0.91–0.95)	<.001
Excluding participants with diabetes	0.90 (0.84–0.97)	.004	0.94 (0.92–0.97)	<.001
Excluding participants with cancer	0.91 (0.85–0.97)	.005	0.95 (0.93–0.97)	<.001
Excluding participants aged <40 years	0.91 (0.85–0.97)	.006	0.95 (0.93–0.97)	<.001
Excluding extreme energy intake (<500 or >5000 kcal/day)	0.92 (0.86–0.98)	.009	0.95 (0.93–0.97)	<.001
Multiple imputation for missing data	0.91 (0.86–0.97)	.003	0.94 (0.92–0.96)	<.001
UKB: outcome restricted to ICD-10 K80	N/A	N/A	0.94 (0.91–0.97)	<.001
UKB: participants with ≥2 WebQ completions	N/A	N/A	0.94 (0.91–0.97)	<.001
NHANES 13-component score (omitting green tea)	0.92 (0.86–0.98)	.009	N/A	N/A
Harmonized 12-component score (no green tea + chickpeas)	0.93 (0.87–0.99)	.023	0.96 (0.94–0.98)	<.001
Per 1-SD increase in DI-GM (standardized)	0.84 (0.79–0.89)	<.001	0.89 (0.87–0.91)	<.001
Excluding participants with prior cholecystectomy	0.93 (0.87–0.99)	.023	0.95 (0.93–0.97)	<.001
Further adjusted for physical activity	0.92 (0.86–0.98)	.009	0.95 (0.93–0.97)	<.001
Women: + parity and OC/HRT use (UKB)	N/A	N/A	0.94 (0.88–1.00)	.053
NHANES: restricted to two-recall participants	0.91 (0.85–0.97)	.003	N/A	N/A
UKB: incident cases only (K80 after diet assessment)	N/A	N/A	0.96 (0.94–0.98)	<.001

Estimates are odds ratios per 1-point increase in DI-GM from the conservative model unless otherwise noted. CI, confidence interval; HRT, hormone-replacement therapy; N/A, not applicable; OC, oral contraceptive; SD, standard deviation; UKB, UK Biobank; WebQ, Oxford Web-based 24-hour dietary questionnaire

Supplementary Table 2. Component-by-component prevalence of DI-GM points in NHANES and the UK Biobank

DI-GM component	% scoring 1 point, NHANES	% scoring 1 point, UK Biobank
Avocado	35.0%	49.9%
Broccoli	37.7%	53.0%
Chickpeas	16.7%	N/A
Coffee	52.0%	50.5%
Cranberries	36.8%	42.5%
Fermented dairy	53.9%	49.0%
Fiber	49.0%	52.2%
Green tea	5.3%	55.0%
Soy	39.1%	48.5%
Whole grains	45.2%	52.9%
High-fat diet	35.5%	50.8%
Processed meat	49.8%	38.9%
Red meat	40.1%	48.3%
Refined grains	51.2%	46.2%

Proportion of participants awarded 1 point on each DI-GM component in each cohort. DI-GM, Dietary Index for Gut Microbiota; N/A, not available

Supplementary Table 3. DI-GM scoring criteria in NHANES and the UK Biobank

Component	Direction	NHANES (M)	NHANES (F)	UKB (M)	UKB (F)	Scoring
Favorable components						
Avocado (g/day)	Favorable	≥0.10	≥0.08	≥0.15	≥0.12	≥median: 1
Broccoli (g/day)	Favorable	≥2.50	≥2.20	≥3.10	≥2.80	≥median: 1
Chickpeas (g/day)	Favorable	≥0.05	≥0.04	N/A	N/A	≥median: 1
Coffee (g/day)	Favorable	≥120.0	≥95.0	≥180.0	≥150.0	≥median: 1
Cranberries (g/day)	Favorable	≥0.30	≥0.25	≥0.20	≥0.18	≥median: 1
Fermented dairy (g/day)	Favorable	≥15.0	≥18.0	≥20.0	≥25.0	≥median: 1
Fiber (g/day)	Favorable	≥16.5	≥14.2	≥18.0	≥15.5	≥median: 1
Green tea (g/day) a	Favorable	≥0.01	≥0.01	≥0.05	≥0.05	≥median: 1
Soy (g/day)	Favorable	≥0.10	≥0.08	≥0.12	≥0.10	≥median: 1
Whole grains (g/day)	Favorable	≥25.0	≥22.0	≥30.0	≥28.0	≥median: 1
Unfavorable components						
High-fat diet (% energy) b	Unfavorable	≥40%	≥40%	≥40%	≥40%	<threshold: 1
Processed meat (g/day)	Unfavorable	≥18.0	≥12.0	≥22.0	≥15.0	<median: 1
Red meat (g/day)	Unfavorable	≥55.0	≥40.0	≥50.0	≥38.0	<median: 1
Refined grains (g/day)	Unfavorable	≥180.0	≥150.0	≥160.0	≥135.0	<median: 1
Total score range		0–14	0–14	0–13 c	0–13 c	

Cutoff values are sex-specific cohort medians (M, male; F, female), except the high-fat component. a Green tea was identified at the FNDDS food-code level in NHANES, allowing it to be scored (range 0–14); the original index omitted it in NHANES. b Per the original index, the high-fat component uses a fixed threshold of ≥40% of energy from fat, not a sex-specific median. c The UK Biobank score ranges 0–13 because chickpea intake is not available in the Oxford WebQ. DI-GM, Dietary Index for Gut Microbiota; N/A, not available.

Supplementary Table 4. Comparison of included versus excluded participants

Characteristic	Standardized difference (NHANES)	Standardized difference (UK Biobank)
Age	0.077	0.033
Sex	0.063	0.019
BMI	0.060	0.038
Current smoking	0.025	0.014
Deprivation index	0.051	0.025
Gallstone prevalence	0.079	0.036

Absolute standardized differences between included and excluded participants were <0.10 in both cohorts, indicating well-balanced analytic samples

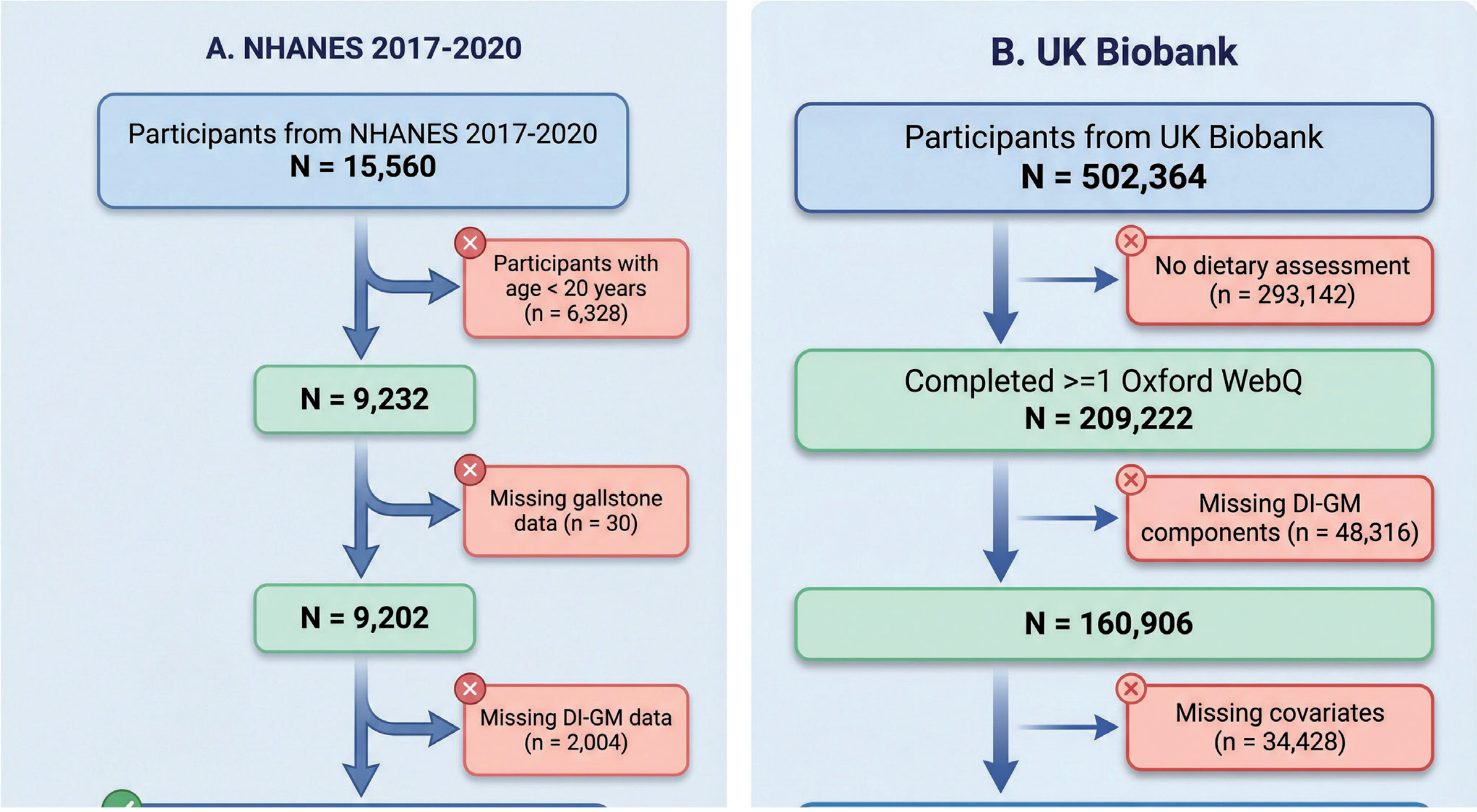

Supplementary Figure 1. Directed acyclic graph for the association between the Dietary Index for Gut Microbiota (DI-GM) and prevalent gallstone disease. Blue: confounders adjusted in the primary total-effect model (Model 2). Yellow: cardiometabolic variables treated as mediators and additionally adjusted only in the conservative model (Model 3); conditioning on them blocks part of the causal path and biases the total effect toward the null. The dashed green arrow denotes the total effect (target estimand). The gut microbiota is unmeasured.
